# Soluble glycoprotein VI, a specific marker of platelet activation is increased in the plasma of subjects with seropositive rheumatoid arthritis

**DOI:** 10.1371/journal.pone.0188027

**Published:** 2017-11-15

**Authors:** John R. Stack, Anne Madigan, Laura Helbert, Eimear Dunne, Elizabeth E. Gardiner, Robert K. Andrews, Roisin Finan, Elizabeth Smyth, Dermot Kenny, Geraldine M. McCarthy

**Affiliations:** 1 Mater Misericordiae University Hospital, Eccles St, Dublin, Ireland; 2 RCSI Molecular & Cellular Therapeutics (MCT), Royal College of Surgeons in Ireland, Dublin, Ireland; 3 ACRF Dept. Cancer Biology and Therapeutics, John Curtin School of Medical Research, Australian National University, Canberra, Australia; 4 Australian Centre for Blood Diseases, Monash University, Melbourne, Australia; University Medical Center Freiburg, GERMANY

## Abstract

**Objectives:**

Anti-citrullinated protein antibodies (ACPA) have been shown to cause platelet activation *in vitro*, through the low-affinity immunoglobulin G (IgG) receptor (FcγRIIa) on platelets. Platelet activation via engagement of FcγRIIa results in proteolytic cleavage and shedding of platelet specific glycoprotein VI (GPVI) which can be detected in the plasma as soluble GPVI (sGPVI). We hypothesized that plasma levels of sGPVI would be increased among patients with seropositive RA as a consequence of antibody-induced platelet activation and GPVI shedding.

**Methods:**

Samples from 84 patients with RA (65 seropositive and 19 seronegative) and 67 healthy controls were collected prospectively and analysed for sGPVI using a standardised ELISA.

**Results:**

Patients with seropositive RA had significantly higher levels of sGPVI compared to seronegative RA and controls. Median (IQR) sGPVI levels were 4.2 ng/ml (3.2, 8.0) in seropositve RA, 2.2 ng/ml (1.5, 3.5) in seronegative RA and 2.2 ng/ml (1.6, 3.4) in controls (p<0.0001). sGPVI levels correlated with ACPA titres (r = 0.32, p = 0.0026) and with RF titres (r = 0.48, p<0.0001).

**Conclusion:**

Plasma sGPVI, a specific marker of platelet activation is increased among patients with seropositive RA.

## Introduction

Arterial thrombosis is a major cause of mortality in rheumatoid arthritis (RA).[1] Patients with RA have an increased risk of cardiovascular disease (CVD) similar to that of diabetes mellitus and have twice the risk of sudden cardiac death when compared to the general population.[2,3] CVD accounts for >50% of deaths among patients with RA.[2] The risk is especially increased for patients who are positive for anti-citrullinated protein antibodies (ACPA) or rheumatoid factor (RF).[4–7] Whilst the reasons for this increased risk remain incompletely understood, recent studies point towards platelet activation and early vascular inflammation as possible mechanisms.

Platelets are well known for their role in thrombosis and haemostasis, however until recently, their inflammatory potential remained less well explored. Platelet activation results in the release of biologically active microparticles [MP] which can promote the expression of a host of inflammatory cytokines.[8] In the setting of thrombosis, enhanced platelet activity results in the release of α-granules containing adenosine diphosphate (ADP), serotonin, P-selectin, chemokines and cytokines.[9] Release of these pro-thrombotic or pro-inflammatory substances ultimately culminates in activation of the platelet-specific integrin αIIbβ3, enhanced platelet aggregation and thrombus formation. Included amongst the array of cytokines released are IL-1, IL-6, IL-8 and TNFα, all of which are known for their pro-inflammatory properties.[8] Excessive platelet activation can result in vascular inflammation, atherosclerotic plaque instability and plaque rupture.[1,2] Platelet activation which has been shown to occur in RA could therefore potentially explain, at least in part, the aggravated cardiovascular disease risk that exists among these patients.

Platelet activation results in proteolytic cleavage and shedding of the platelet specific glycoprotein VI receptor (GPVI).[10] GPVI is a ~64-kDa transmembrane glycoprotein which is found exclusively on platelets and megakaryocytes, and acts as the predominant platelet receptor for collagen. It contains 2 extracellular immunoglobulin (Ig)- like domains, a mucin domain, transmembrane domain and a cytoplasmic tail of approximately 50 residues. GPVI is non-covalently associated with the Fc receptor γchain (FcRγ) via an intramembrane salt bridge and signals via immunoreceptor-tyrosine based active motifs (ITAM)s within the cytoplasmic tail portion of dimerized Fcγ chains. Signal transduction occurs when ligand engages with GPVI resulting in phosphorylation of the ITAM motif and proteolytic cleavage of the GPVI receptor at the platelet surface. A 55-kDa ectodomain fragment, soluble GPVI (sGPVI) is shed leaving behind a 10-kDa remnant. Shedding is rapid and irreversible and is likely to function as a limiting step in collagen-mediated platelet adhesion and thrombin generation.[10] GPVI shedding can however also be precipitated by a variety of other mechanisms including engagement of the low affinity immunoglobulin G (IgG) receptor (FcγRIIa) on platelets, which also utilises ITAM signalling.[11]

Importantly, sGPVI has been shown to be a useful biomarker of platelet activation as it can be measured using a simple ELISA assay and levels are not influenced by age, gender or smoking status.

Recently it was shown that ACPA can cause platelet activation *in vitro* via FcγRIIa.[12] Given that ligand binding to FcγRIIa results in activation of the GPVI shedding pathway, we hypothesized that levels of sGPVI would be increased among patients with seropositive RA as a consequence of antibody induced platelet activation and GPVI shedding.

## Methods

### Subjects

All subjects provided written informed consent to participate in the study. The study was approved by the research ethics committee of the Mater Misericordiae University Hospital (MMUH) and the Royal College of Surgeons of Ireland (RCSI). 84 patients with RA (65 seropositive and 19 seronegative) were recruited prospectively at the MMUH, from 2012 until 2016. They satisfied both the 1987 American College of Rheumatology (ACR) and the 2010 ACR/European League Against Rheumatism (EULAR) criteria. Controls (n = 67) comprised of 17 patients with osteoarthritis also attending the MMUH and 50 healthy volunteers working at the RCSI. The study was conducted according to the principles expressed in the Declaration of Helsinki. Demographic and clinical data were collected for all participants. Characteristics of seropositive vs seronegative RA are presented in Table 1.

**Table 1:** Characteristics of patients with seropositive RA vs seronegative RA.

	Sero + RA	Sero—RA	*P* value
Total Number	65	19	
Female, n (%)	51 (78)	16 (84)	ns
Male, n (%)	14 (21)	3 (15)	ns
Age, yr (median [IQR])	62 [54–71]	48[38–64]	0.01
CRP, mg/l (median [IQR])	8 [4–15]	6 [2–21]	ns
ESR, mm/hr (median [IQR])	21 [11–32]	13 [7–38]	ns
WCC x 10^9^/L (median [IQR])	7.5 [5.6–9.0]	7.16 [5.8–8.3]	ns
Monocyte x10^9^/L (mean +/- SEM)	0.47 +/- 0.02	0.47 +/- 0.03	ns
Fibrinogen g/l (mean +/- SEM)	3.33 +/- 1.12	4.06 +/- 1.92	ns
Platelet Count x 10^9^(mean +/- SEM)	281+/- 98	285 +/- 81	ns
DAS28-CRP (Mean +/- SEM)	3.83 +/- 1.49	4.37 +/- 2.2	ns
RF titre, IU/mL (median [IQR])	81 [20–271]	2.3 [1.5–4.7]	<0.0001
CCP titre, u/mL (median [IQR])	212 [100–340]	2.1 [1.1–3.6]	<0.0001
Aspirin usage, n (%)	10 (15)	1 (5)	ns
CAD/ Thrombotic event*, n (%)	6 (9)	0 (0)	ns
TNF inhibitor usage, n (%)	22 (35)	6 (31)	ns

*RA* rheumatoid arthritis, *CRP* c- reactive protein, *ESR* erythrocyte sedimentation rat*e*, *WCC white cell count*, *DAS28* Disease Activity Score in 28 joints, *RF rheumatoid factor*, *CCP citrullinated c-protein*, *CAD coronary artery disease*

* defined as: *myocardial infarction*, *stroke or percutaneous angioplasty*, *IQR* interquartile range, *SEM* standard error of the mean, ns not significant.

### Blood sampling and plasma preparation

Blood was collected into vacutainers containing 0.106 nM sodium citrate as anticoagulant (10% vol/vol). Platelet poor plasma was prepared by centrifugation of whole blood at 2000g for 10 minutes. Plasma was aliquoted and stored -80°C until analysis.

### Measurement of soluble GPVI

sGPVI levels were measured by immunoassay. 96 well standard binding plates from MesoScale Discovery (MSD, Rockville, MD) were coated overnight at 4°C with 4 μg/mL sheep anti-human GPVI polyclonal antibody (R&D Systems, Abingdon, UK). The plate was blocked with 5% MSD Blocker A for 1 hour at RT, washed x3 with 150 μL PBS / 0.05% Tween (PBST) and 25 μL of undiluted platelet poor plasma added to duplicate wells. Samples were incubated at RT with vigorous shaking for 1 hour. The plate was washed x3 with PBST. Biotinylated sheep anti-human GPVI antibody was diluted to 1 μg/mL in 1% MSD Blocker A and 25 μL added to each well. The plate was incubated at RT for 1 hour with shaking at 650 rpm then washed x3 with PBST. 150 μL 2x read buffer was added to each well and the plate read on a MesoScale Quickplex SQ120 Plate Scanner according to the manufacturer’s instructions. The intra- and inter-assay coefficients of variation (CVs) for sGPVI were 4.9 and 5% respectively. References ranges for sGPVI among healthy individuals have been reported previously. [13] A full description of the assay has previously been published. [14]

### Statistical analysis

The Kolmogorov-Smirnov test was used to determine whether data sets were parametric or non-parametric. Results were expressed as mean +/- standard deviation (SD) or median +/- interquartile range (IQ) depending on whether they were derived from parametric or non-parametric data respectively. Mann-Whitney U test and Kruskal–Wallis test was used to compare groups. Spearman’s Rank Correlation Coefficient was used to assess for associations between sGPVI levels and demographic and clinical markers. GraphPad Prism Version 6.05 was used for data analysis.

## Results

Patients with seropositive RA were significantly older, but no signficant correlation was observed between levels of sGPVI and age, CRP, fibrinogen, ESR, platetet count or DAS28-CRP (Table 1). In total there were 13 patients in whom a sGPVI level >10ng/L was detected. Patients with a sGPVI >10ng/ml were older than patients with a sGPVI level <10ng/ml (65.3+/- 3.2 vs 57.3+/-1.6 respectively) however this did not reach statistical significance (p = 0.055). Similarly no signficant differences were observed between antibody status and use of anti-platelet medication or TNF inhibitors.

The frequency of thrombotic events/ CAD was higher in the ACPA positive RA group compared to the ACPA negative group (6 vs 0) however this did not reach significance. The median sGPVI level among patients in which an event had occurred was 4.52 ng/ml vs 3.79 ng/ml in the event free group however this was also not statistically significant (p = 0.572).

**Fig 1:**
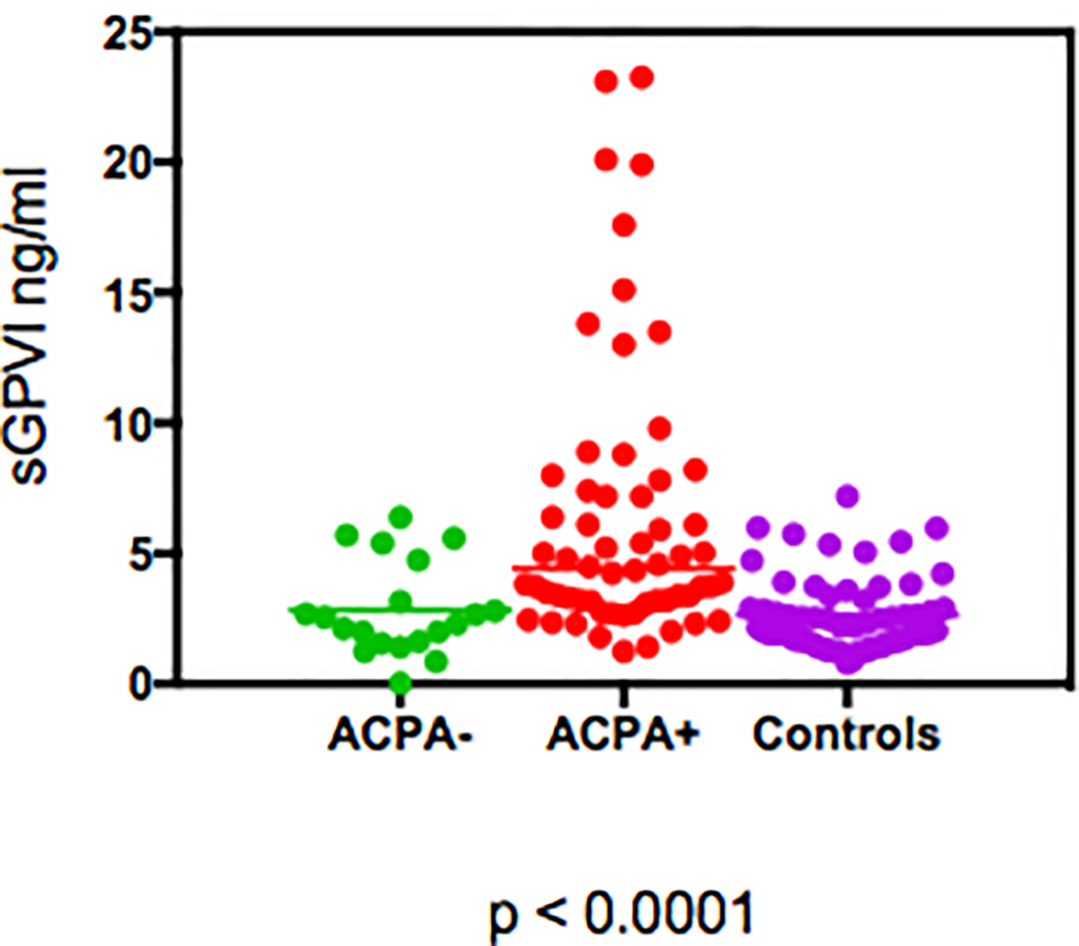
Levels of sGPVI in patients with seropositve RA vs seronegative RA vs controls.

Patients with seropositive RA had significantly higher levels of sGPVI compared to seronegative RA and controls (Fig 1). Median (IQR) sGPVI levels were 4.2 ng/ml (3.2, 8.0) in seropositve RA, 2.2 ng/ml (1.5, 3.5) in seronegative RA and 2.2 ng/ml (1.6, 3.4) in controls (p<0.0001). sGPVI levels correlated with ACPA titres (r = 0.32, p = 0.0026) and also with RF titres (r = 0.48, p<0.0001).

## Discussion

This is the first *in vivo* study to identify an association between sGPVI, a marker of platelet activation and seropositive RA in humans. Platelet activation in RA was first described by Boilard et al who showed that platelet MPs were present in abundance within RA synovial fluid (SF), and were capable of inducing cytokine release from synovial fibroblasts.[15] Using a K/BxN serum transfer mouse model of RA they went on to demonstrate that platelet depleted mice displayed a markedly reduced inflammatory phenotype. Using a combination of pharmacological blockade and *Gp6* ^*-/-*^ mice they further showed that signaling via GPVI was the predominant pathway through which platelet MP generation was mediated.

More recently it was demonstrated by Habets et al that platelet activation is associated with the presence of ACPA and that blockade of the low affinity platelet IgG receptor, FcγRIIa *in vitro*, inhibited ACPA-mediated platelet activation.[12] FcγRIIa was therefore identified as the ligand through which ACPA-mediated platelet activation occurs. ITAM-based signalling via engagement of either GPVI or FcγRIIa results in shedding of GPVI.[11] This mechanism is not unique to ACPA and has been demonstrated in other diseases such as heparin-induced thrombocytopenia (HIT) and immune mediated thrombocytopenia (ITP) where anti-platelet antibodies derived from patients with either HIT or ITP were able to activate GPVI shedding via FcγRIIa engagement.[11,16,17] Similarly, circulating immune complexes derived from patients with systemic lupus erythematosis (SLE) have been shown to activate platelets *in vitro* via FcγRIIa.[18] In a separate study, anti-GPVI IgG antibodies were detected in a patient with SLE resulting in impaired platelet response to collagen.[19] We hypothesise that a similar ‘dual signaling’ mechanism is involved in seropositive RA whereby ACPA and/or RF can mediate platelet activation and where sGPVI is a quantifiable measure of this process.

In this study both ACPA and RF correlated with sGPVI. The vast majority of seropositive RA patients were positive for both ACPA and RF antibodies therefore we were unable to distinguish whether the association bewteen sGPVI and seropositive RA was due to the presence of ACPA, RF or both. Although platelets lack the Fc-IgM receptor, RF factor can exist in both IgM and IgG form so conceivably RF-IgG could potentially engage with platelets. Interestingly only one patient in the seropositive RA group had undetectable ACPA but was strongly RF positive. This patient expressed high levels of sGPVI suggesting that the presence of RF alone could lead to shedding of GPVI. However we cannot draw any firm conclusions from this single observation.

Our study has a number of caveats. We did not directly show that elevated sGPVI in our patients was due to FcγRIIa-mediated pathways and our approach to investigating platelet activation was single-faceted. We did not examine for levels of other known platelet agonists such as TNFα which has also been shown to be involved in platelet activation,[20,21] nor did we compare sGPVI with other measures of platelet activation (eg plasma soluble P-selectin) that could have further defined the degree of platelet activity in patients recruited to the study.[22] However, previous studies have illustrated significant correlation between plasma sGPVI and soluble P-selectin. Furthermore, sGPVI has been shown to have advantages over P-selectin in terms of selectivity, specificity and age-dependence.[10,22] We have previously demonstrated that patients with RA have a decreased response to collagen induced platelet aggregation as well as increased levels of sGPVI, which supports the hypothesis that enhanced activation of the GPVI pathway occurs in patients with RA. [23]. Future studies of an expanded population should permit further concurrent correlation of platelet function and plasma concentrations of sGPVI.[23]

It is interesting to note that a similar association has been observed between sGPVI and acute ischaemic stroke. Within this study we retrospectively identified an increased number of thrombotic events within the ACPA positive group who tended to have higher levels of sGPVI however this did not reach significance. It is possible that we may not have captured all thrombotic events, as some events may have occurred at a different institution and not been recorded in our electronic patient record system. Whilst not statistically significant there was a 3–fold increase in aspirin usage among the seropositive RA group. This raises the question as to whether aspirin could have influenced the increased levels of sGPVI that were observed within this group. There is a paucity of data in the literature examining the effects of aspirin on GPVI expression however one recent study showed that aspirin had no effect on GPVI exosome secretion in-vitro.[24] This would suggest that aspirin does not influence GPVI cleavage however further studies are needed to corroborate this finding. It is possible that the increased use of aspirin in the seropositive RA group simply represents a surrogate for established cardiovascular disease within this population. It would be of interest to study a group of RA patients prospectively to see whether sGPVI could be predictive of future thrombotic events.

Lastly there are studies that indicate that blockade of GPVI function may help to reduce the risk of thrombosis without increasing the risk of bleeding.[9,21] It is currently unknown what effect using such targeted therapies would have in RA. However given the attenuated effects of inflammatory arthritis that are seen using GPVI blockade in mice, targeting of GPVI in RA in humans may be beneficial and warrants further investigation.

## Supporting information

S1 Table Original data set. (XLSX)
